# Genomic study substantiates the intensive care unit as a reservoir for carbapenem-resistant Klebsiella pneumoniae in a teaching hospital in China

**DOI:** 10.1099/mgen.0.001299

**Published:** 2024-09-26

**Authors:** Shuo Jiang, Zheng Ma, Huiluo Cao, Li Mo, Jinlan Jin, Bohai Yu, Kankan Chu, Jihua Hu

**Affiliations:** 1 Shenzhen Hospital (Futian) of Guangzhou University of Chinese Medicine, 6001 Beihuan Avenue, Shenzhen, Guangdong, PR China, Shenzhen, Guangdong, PR China; 2 Department of Microbiology, University of Hong Kong, Hong Kong, PR China

**Keywords:** antimicrobial resistance, Carbapenem-resistant *Klebsiella pneumoniae* (CRKP), hospital transmission, intensive care unit (ICU)

## Abstract

Carbapenem-resistant *Klebsiella pneumoniae* (CRKP) has recently emerged as a notable public health concern, while the underlying drivers of CRKP transmission among patients across different healthcare facilities have not been fully elucidated. To explore the transmission dynamics of CRKP, 45 isolates were collected from both the intensive care unit (ICU) and non-ICU facilities in a teaching hospital in Guangdong, China, from March 2020 to August 2023. The collection of clinical data and antimicrobial resistance phenotypes was conducted, followed by genomic data analysis for these isolates. The mean age of the patients was 75.2 years, with 18 patients (40.0%) admitted to the ICU. The predominant strain in hospital-acquired CRKP was sequence type 11 (ST11), with k-locus type 64 and serotype O1/O2v1 (KL64:O1/O2v1), accounting for 95.6% (43/45) of the cases. The CRKP ST11 isolates from the ICU exhibited a low single nucleotide polymorphism (SNP) distance when compared to isolates from other departments. Genome-wide association studies identified 17 genes strongly associated with SNPs that distinguish CRKP ST11 isolates from those in the ICU and other departments. Temporal transmission analysis revealed that all CRKP isolates from other departments were genetically very close to those from the ICU, with fewer than 16 SNP differences. To further elucidate the transmission routes among departments within the hospital, we reconstructed detailed patient-to-patient transmission pathways using hybrid methods that combine TransPhylo with an SNP-based algorithm. A clear transmission route, along with mutations in potential key genes, was deduced from genomic data coupled with clinical information in this study, providing insights into CRKP transmission dynamics in healthcare settings.

**Impact Statement**

The infection caused by carbapenem-resistant *Klebsiella pneumoniae* (CRKP) has recently emerged as a substantial public health concern. From March 2020 to August 2023, 45 CRKP strains were collected from multiple facilities in a teaching hospital in China. Predominantly, the hospital infections were caused by CRKP isolates of sequence type 11 (ST11) with k-locus type 64 (KL64) carrying *bla*_KPC-2_ gene. This study demonstrated that CRKP-ST11 isolates from patients in non-ICU departments were genetically highly similar to those from ICU patients. We further identified single nucleotide polymorphism (SNP) discrepancies between isolates from ICU and non-ICU wards. A patient-to-patient transmission route was clearly illustrated across departments within a single hospital. This study elucidates a clear transmission route for CRKP, along with mutations in key genes, providing valuable insights into infection control within healthcare settings.

## Data Summary

All the raw reads generated in this study were deposited in the National Centre for Biotechnology Information (NCBI) under Bioproject ID PRJNA1085970. All clinical metadata can be accessed in Tables S1 and S2 (available in the online version of this article).

## Introduction

*Klebsiella pneumoniae* is a Gram-negative bacterium of significant clinical importance, particularly in China [1]. High mortality rates were observed in patients with carbapenem-resistant *K. pneumoniae* (CRKP) infection, especially those in intensive care units (ICUs) [2]. The occurrence of CRKP infections in healthcare settings, the environment, and among healthcare workers have been consistently reported [3,4]. The primary mechanism for carbapenem resistance in *K. pneumoniae* involves the production of carbapenemases, such as *Klebsiella pneumoniae* carbapenemase (KPC) and CRKP has been listed as a significant pathogen by the World Health Organization (WHO), underscoring the need for stringent infection control measures and precise treatment strategies. In Asia, the most prevalent clone of carbapenem-resistant *K. pneumoniae* is the *bla*_KPC-2_-positive sequence type (ST) 11, accounting for a significant majority of CRKP cases, ranging from 60–70% in China [5]. Recent studies have shown that ST11 strains with acquired hypervirulence have emerged as the most widespread population of CRKP in China, representing a growing high-risk clone [6,8].

Nosocomial CRKP infections frequently occur in ICUs due to the presence of immunocompromised patients and the high consumption of antibiotics. In China, ICUs are designed for precise treatment and monitoring, typically consisting of a large room with separate areas for each patient [2]. The confined space within ICUs promotes the circulation and spread of CRKP within the hospital [9]. In view of the virulence of CRKP, improving infection control measures, such as hand hygiene and environmental cleaning, is crucial for preventing the transmission of CRKP in healthcare settings. Many studies have been conducted to investigate outbreaks within ICUs in China [10,11], while the CRKP transmission among patients from different facilities remains poorly understood. Therefore, obtaining a thorough understanding of CRKP transmission at multiple levels could prove valuable in optimizing surveillance efforts.

In this study, CRKP isolates were collected in different departments within the hospital. The occurrence of CRKP isolates was monitored from March 2020 to August 2023. We investigated CRKP strains in a teaching hospital in Guangdong, China, with the aim of obtaining a clear understanding of the genomic characteristics and transmission of CRKP strains.

## Methods

### Collection of *K. pneumoniae* clinical isolates

All CRKP isolates were collected in a leading hospital in Guangdong, China with 1080 beds across 37 wards. CRKP isolates were collected from two sources: 1) clinical specimens obtained during routine diagnostic procedures and 2) anal swabs collected through a proactive screening programme targeting patients upon hospital admission. The proactive screening programme focused on patients meeting the following criteria: adults (≥18 years old) with an anticipated hospital stay exceeding 48 h who were either admitted to the ICU or classified as high-risk for CRKP acquisition. High-risk status was determined according to the World Health Organization (WHO) guidelines for the prevention and control of carbapenem-resistant Enterobacteriaceae in healthcare facilities [12]. Specifically, patients were considered high-risk if they met any of the following criteria: history of CRKP colonization or infection, epidemiological linkage to a CRKP-infected patient, prior hospitalization in a region with high CRKP prevalence, immunosuppression and organ transplantation. When multiple CRKP isolates with identical susceptibility profiles were obtained from a single patient, only the first isolate collected was included in the genomic analysis.

We defined CR-hvKP as a CRKP strain that demonstrates the presence of the combination of *rmpADC*/*rmpA2* with *iucA*, *iroB,* or *peg-344* and exhibits hypermucoviscosity (HM-positive) as previously described [13,15]. For this study, the definition of healthcare-associated infection (HAI) was a KP infection acquired by a patient who was either hospitalized for more than 48 h or had any interaction with the healthcare system within the preceding 90 days in the first 48 h of hospitalization. Community-acquired infection (CAI) was defined as admission within 48 h, and individuals had no contact with healthcare facilities within the 90 day period, as previously described [16]. The clinical information of the patients with sample collection date, sample type, admission department, demographic characteristics, underlying disease, and outcomes, was collected. All the isolates collected from multiple sources were identified by a Clin-TOF II (Bioyong, Beijing, China) and tested for carbapenem resistance following the breakpoints of the Clinical and Laboratory Standards Institute (CLSI-M100, 33rd edition) guidelines [17]. All the selected isolates were stored in the MicroBank (Biocaring, Guangdong, China) at −80 °C until further analysis.

This research received ethical approval from the Ethics Committee of Shenzhen Hospital of Guangzhou University of Chinese Medicine (KS-2021053–1). Consent was obtained from all participants before the project, and all personal data were anonymized.

### Antimicrobial susceptibility testing

The minimum inhibitory concentration (MIC) was tested by VITEK 2 Compact (BioMérieux, Marcy l'Etoile, Lyon, France) using Clinical and Laboratory Standards Institute (CLSI) guideline breakpoints [17]. The antibiotics included imipenem, ertapenem, amikacin, cefepime, levofloxacin, piperacillin/tazobactam, ceftriaxone, cefoperazone/sulbactam, cotrimoxazole, cefuroxime, amoxicillin/clavulanic acid, cefoxitin, ceftazidime and meropenem. Resistance to three or more classes of antimicrobial agents was defined as multidrug resistance (MDR) [18].

### Detection of hypermucoviscosity

*Klebsiella pneumoniae* strains were cultured on Columbia 5% sheep blood agar (Detgerm, Guangzhou, China) and incubated at 37 °C overnight. The formation of viscous strings was observed by lightly touching the surface of individual colonies using a standard bacterial inoculation loop. If the produced viscous strings exceeded a length of 5 mm, the strains were classified as HM-pos, as previously described [19].

### Genomic DNA extraction and sequencing data analysis

Bacterial genomic DNA (gDNA) was extracted with a TIANamp bacteria genomic DNA kit (TIANGEN BIOTECH, Beijing, China). Whole-genome sequencing was performed using a Novaseq 6000 (Illumina, San Diego, United States) and a QuarPrep EZ DNA library kit (Dynegene, Shanghai, China) was used for library preparation. All short reads were subjected to quality control using FastQC [20] and *de novo* assembly was conducted by SPAdes v3.15.3 [21] using *k*-mer sizes of 21, 33, 55, 77, 99, and 127 with the --careful flag. Then, assembly statistics and quality assessment were performed using seqkit v2.6.0 [22] and checkM v1.16 [23].

Kleborate v2.3.1 [24] and Kaptive v2.0.7 [25] were used to analyse the bacteria *in silico* for multilocus sequence typing (MLST), antibiotic resistance genes, capsule locus type, and virulence factors. PlasmidFinder v2.1.6 [26] was used to predict the potential plasmid, and the formula for the plasmid replicon type based on the plasmid PubMLST scheme [27] was assigned for each genome. In order to identify SNPs associated with the ICU and non-ICU strains, we used pyseer v1.3.10 [28] to assign significant SNPs to bacterial factors. All significant SNP-associated proteins were annotated using BLASTP against the database of clusters of orthologous genes (COGs) and the NCBI protein database, with a threshold of 80% identity and coverage, and an E-value of less than 1e^−10^.

The *K. pneumoniae* strain HS11386 (accession number: CP003200.1) was selected as the reference genome for the phylogenetic analysis due to its relevance to the isolates in our study. This strain, belonging to ST11 and carrying the *bla*_KPC-2_ gene, was initially isolated from a sputum specimen in China in 2011. We mapped all *K. pneumoniae* isolates in this study to the reference using Snippy v4.6.0 (https://github.com/tseemann/snippy) and the recombination sites were removed using Gubbins v2.4.1 [29]. A maximum-likelihood phylogenetic tree was constructed using iqtree v2.2.0.3 [30] based on clean core genome alignments. The isolate transmission analysis used a threshold-based approach based on SNPs less than or equal to 16 as previously described [31,32]. In addition, automated inference of person-person transmission routes from the genomic data was estimated using TransPhylo [33].

### Statistics analysis and visualization

The Chi-squared test and Wilcoxon test were used where appropriate. Plots were generated using the ggplot2 package (https://ggplot2.tidyverse.org/). A level of significance at 5% was adopted. Cytoscape v3.9.1 was used to construct the genome transmission networks [34]. The phylogenetic trees were visualized and annotated using iTOL v6 [35].

## Results

### Bacteria collection

A total of 616 *K*. *pneumoniae* isolates were collected among 409 patients across 15 departments, originating from clinical specimens. Of these, 126 (20.5 %) isolates were identified as carbapenem-resistant. The ICU yielded the highest number of CRKP isolates from clinical specimens (*n*=51), with other departments each contributing fewer than 20 isolates (Table 1) . Proactive screening, utilizing anal swabs, was conducted on 1466 patients, identifying nine CRKP-positive individuals carrying a total of 29 CRKP isolates. The majority of these isolates (*n*=26) originated from ICU patients, with the remaining three isolates recovered from patients in the cardiovascular disease (CVD) department. To avoid redundancy, in instances where multiple CRKP isolates with indistinguishable susceptibility patterns were recovered from the same patient, only the initial isolate was selected for genomic analysis. This resulted in a final cohort of 45 unique CRKP isolates. These isolates were recovered from patients in 11 out of the 15 departments, with no CRKP isolates detected in patients from the departments of surgery, gynaecology and obstetrics, orthopaedics, or paediatrics.

**Table 1.** Distribution of CRKP isolates from clinical specimens and proactive screening across hospital departments (2020–2023)

Department	Clinical specimens			Proactive screening			Unique CRKP
	No. of patients with KP	No. of KP	No. of CRKP	No. of patients	No. of patients with CRKP	No. of CRKP	
CVD	19	29	5	26	1	3	2
Emergency	6	8	2	16	0	0	1
Encephalology	20	34	11	87	0	0	4
Endocrinology	8	9	2	15	0	0	1
Gastroenterology	5	8	2	29	0	0	1
ICU	107	180	51	562	8	26	18
Massage	4	6	1	35	0	0	1
Nephrology	66	84	17	107	0	0	5
Oncology	54	66	15	227	0	0	5
Pulmonology	38	52	10	190	0	0	3
Recovery	36	54	10	102	0	0	4
Surgery	20	46	0	12	0	0	0
Gynaecology and obstetrics	8	12	0	0	0	0	0
Orthopaedics	8	13	0	28	0	0	0
Paediatrics	10	15	0	30	0	0	0
Total	409	616	126	1466	9	29	45

CRKP, carbapenem-resistant *Klebsiella pneumoniae*CVD, cardiovascular disease; ICU, intensive care unit

### Clinical characteristics of patients with *K. pneumoniae* infection

**Fig. 1.**
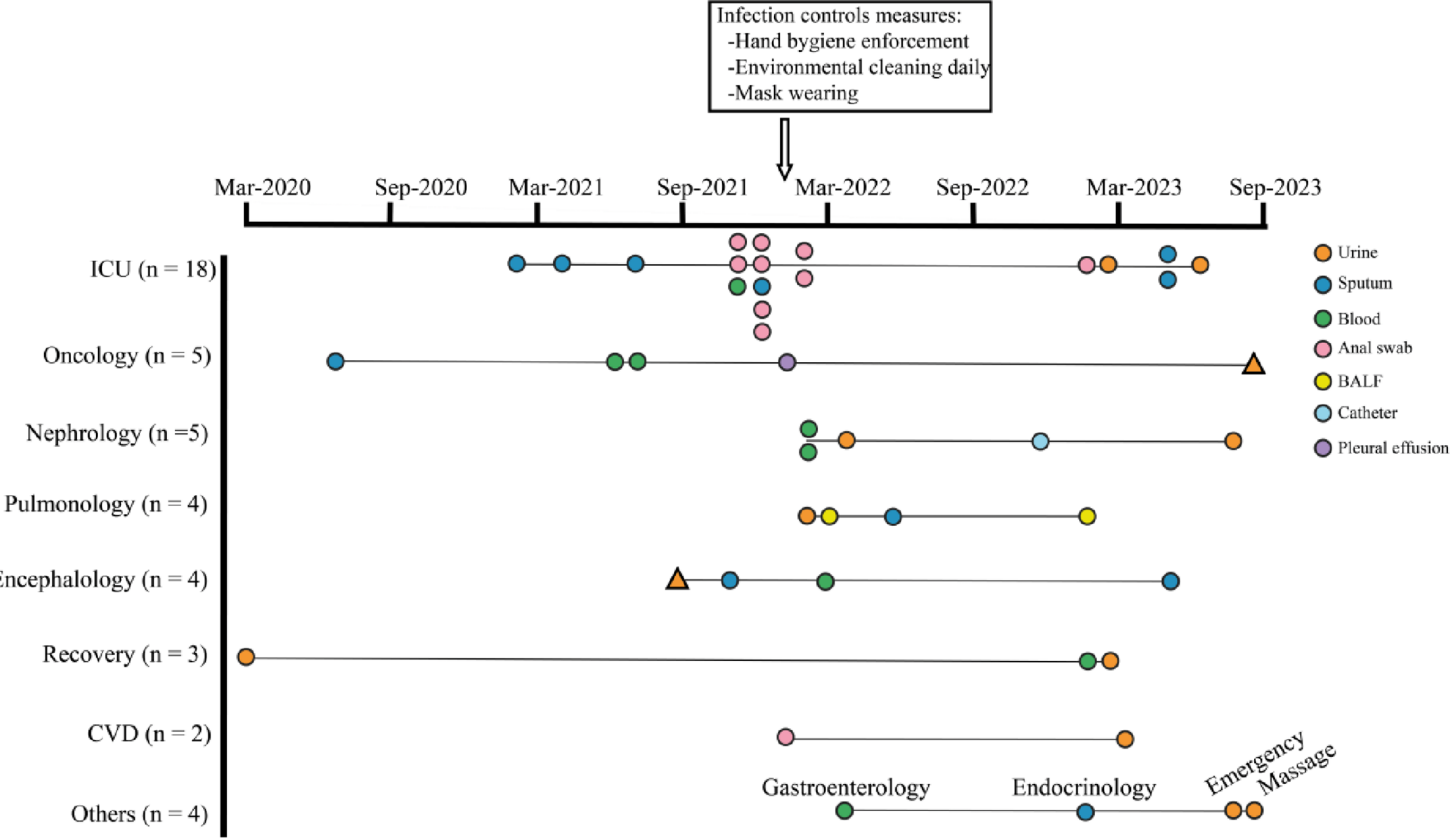
Flowchart representing the timeline of patient enrolment according to different departments. The horizontal lines represent the enrolled departments. Each node represents a patient, and the colour filled in the node indicates the specimen from the patient. The patients infected with non-ST11 CRKP are labelled as triangular nodes. ICU, intensive care unit; CVD, cardiovascular disease; BALF; bronchoalveolar lavage fluid.

A total of 45 patients with *K. pneumoniae* infection were included from March 2020 to August 2023. Twenty-eight patients (62.2%) were males, and the average age of the enrolled patients was recorded as 75.2 years. Specifically, 18 patients (40%, 18/45) were admitted to the ICU. There was an equal proportion of patients in both the oncology and nephrology departments (11.1%), with each department having five out of the 45 patients (Fig. 1). Furthermore, forty-three patients (95.6%, 43/45) were healthcare-associated and only patients 3798 and 4119 with symptoms of pneumonia were community-acquired (Table S1). Among these patients, according to the isolate origin, the most common infection was pneumonia (*n*=15, 33.3%), followed by urinary tract infection (UTI) (*n*=14, 31.3%), intestinal infection (*n*=9, 20%) and bacteraemia (*n*=7, 15.6%) (Table S1). Patients with cardiovascular disease (CVD) accounted for the majority of patients (84.4%, 38/45). More than half of the patients had pulmonary disease (62.2%, 28/45) or urinary disease (51.1%, 23/45).

Based on the results of antimicrobial susceptibility testing, all the isolates were multidrug-resistant. In addition to carbapenem resistance, all the CRKP strains exhibited high resistance pattern to ceftazidime (100%, 45/45), cefepime (100%, 45/45), and amikacin (91.1%, 41/45). Twenty strains (44.4%) exhibited resistance to polymyxin with especially six isolates demonstrating colistin resistance (Table S1). More specifically, all *K. pneumoniae* isolates in this study were resistant to cefoxitin, amoxicillin/clavulanate, and cefuroxime.

### Genomic features of the CRKP isolates

**Fig. 2.**
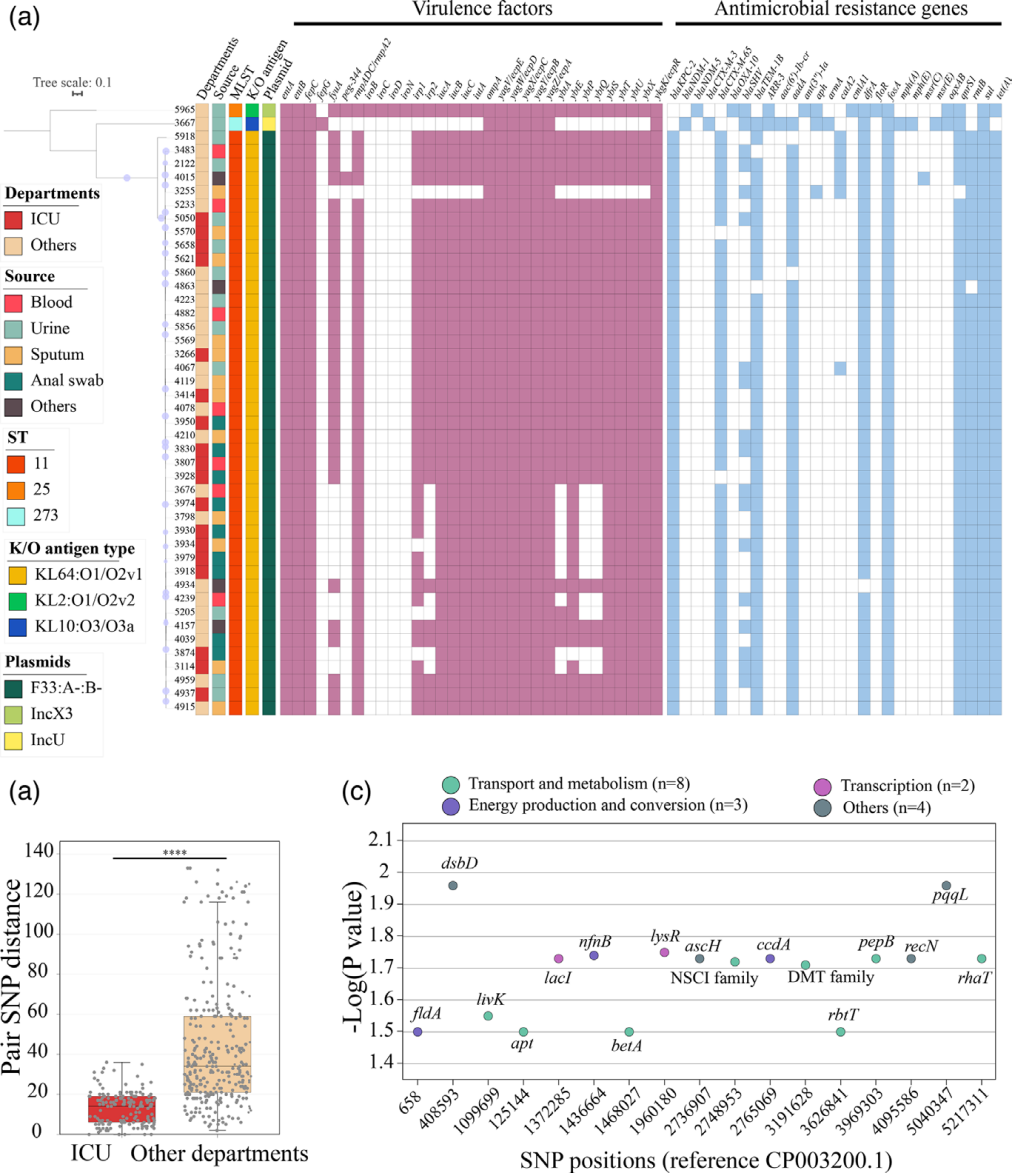
Phylogenetic and comparative genomic analysis of 45 CRKP isolates. (**a**) Maximum-likelihood phylogenetic tree constructed from core genome SNPs of the 45 CRKP isolates. Circle size on each branch corresponds to bootstrap values, ranging from 80 to 100. Metadata and genetic features are displayed alongside the tree. (**b**) Pairwise SNP distance comparison between CRKP ST11 isolates recovered from the ICU and those from other departments. The box in the boxplot represents the interquartile range, encompassing the central 50% of the data, with the median indicated by the central line. Statistical significance was assessed using the Wilcoxon test, where **** denotes *P*<0.0001. (**c**) Genes associated with SNPs exhibiting significant differences in prevalence between CRKP ST11 isolates from the ICU and other departments, compared against the reference genome CP003200.1. ICU, intensive care unit; KL, K-locus; ST; sequence type; SNP, Single nucleotide polymorphism.

A total of 45 *K*. *pneumoniae* isolates were tested by whole-genome sequencing. A phylogenetic tree based on 34230 core SNPs identified the 45 *K*. *pneumoniae* isolates (Fig. 2a). Most of the isolates (95.6%, 43/45) were identified as the ST11 and KL64:O1/O2v1 serotype (Table S2). The remaining two strains were identified as KL2:O1/O2v2 ST25 and KL10:O3/O3a ST273, respectively. The ST11 isolates in this study shared a highly identical profile of virulence factors, including enterobactin (*entAB*), mucoid regulator (*rmpADC*/*rmpA2*), yersiniabactin (*irp1*/*2* and *ybt* gene cluster), iron transporter (*iucABC*) and adhesion (*yag*/*ecp* gene cluster). Only one ST25 strain with the antigen type KL2:O1/O2v2 isolated from the urine sample was associated with both *iroB* and *peg-344*.

All the ST11 isolates were positive for *bla*_KPC-2_ while the remaining two isolates ST25 and ST273, were found to be positive for *bla*_NDM-1_ and *bla*_NDM-5_, respectively. Based on the plasmid replicon typing, all the isolates carried the IncF plasmid with the F33:A-:B- formula, which was strongly associated with *bla*_KPC-2_ dissemination. Most of the *bla_CTX-M_*-positive strains (91.1%, 41/45) were associated with *bla*_CTX-M-65_ and were distributed among the ICU CRKP (*n*=16) and non-ICU CRKP (*n*=29) strains. The isolate from patient urine was *bla*_CTX-M-3_-positive and belonged to the ST273 group. Another common extended-spectrum beta-lactamase, *bla*_TEM-1B_ was detected among the 43 CPKP isolates (95.6%). In addition to the carbapenemase-encoding genes, all the CRKP isolates were found to carry the *fosA* and *sul* genes. Furthermore, 44 strains (97.8%) also possessed the *tet*(A) gene. The common genetic feature of CR-hvKP was the presence of KL64:O1/O2v2-*iucA-rmpADC*/*rmpA2-bla*_KPC-2_-*bla*_TEM-1B_.

Pairwise SNP analysis of CRKP ST11 isolates revealed a greater degree of genetic diversity among isolates from other departments (range: 2–133 SNPs, mean=43.9, SD=31.5) compared to those isolated from the ICU (range: 0–36 SNPs, mean=13.1, SD=8.1). Bacterial genome-wide association studies identified 17 genes exhibiting a strong association with SNPs that differentiated CRKP ST11 isolates from the ICU and other departments (Fig. 2b). The majority of these genes (*n*=8) were implicated in transporter and metabolic functions, including *livK*, *apt*, *betA*, a gene encoding an NSCI family protein, a gene encoding a DMT family protein, *araJ*, *pepB*, and *rhaT*. In addition to transport and metabolism-related genes, mutations in genes involved in transcription and replication also differed between CRKP isolates from these two sources.

### Inference of the transmission route of CRKP isolates

Transmission analysis was performed on 43 CRKP ST11 KL64:O1/O2v1 strains. No putative transmission events with ≤16 SNPs between isolates were observed among the two non-ICU isolates recovered in 2020 (Table 2) . Within the ICU, a high number of potential transmission events were observed in 2021, with numerous isolate pairs (*n*=11) exhibiting a close genetic relationship. In 2022, ten CRKP isolates were recovered from patients in non-ICU departments. However, the predicted transmission network suggests that these isolates were largely unconnected, with only two isolates from the ICU implicated in eight transmission events, linking them to isolates from other departments. The highest number of interdepartmental transmission events (*n*=17) occurred in 2023, involving the exchange of isolates between the ICU and other departments. In contrast, fewer transmission events were observed within the ICU (*n*=6) or solely within other departments (*n*=5) during this period.

**Table 2.** Summary of temporal transmission of 43 CRKP-ST11 across departments between 2021 and 2023

	No. of CRKP ST11		No. of predicted CRKP ST11 transmission			
Year	CRKP from ICU	CRKP from other departments	Within-ICU	ICU-other departments	Other departments	Total transmission
2020	0	2	0	0	0	0
2021	11	3	37	18	1	56
2022	2	10	1	8	6	15
2023	5	10	6	17	5	28
Total	18	25	44	43	12	99

CRKPcarbapenem-resistant *Klebsiella pneumoniae*ICUIntensive care unitSTSequence type

**Fig. 3.**
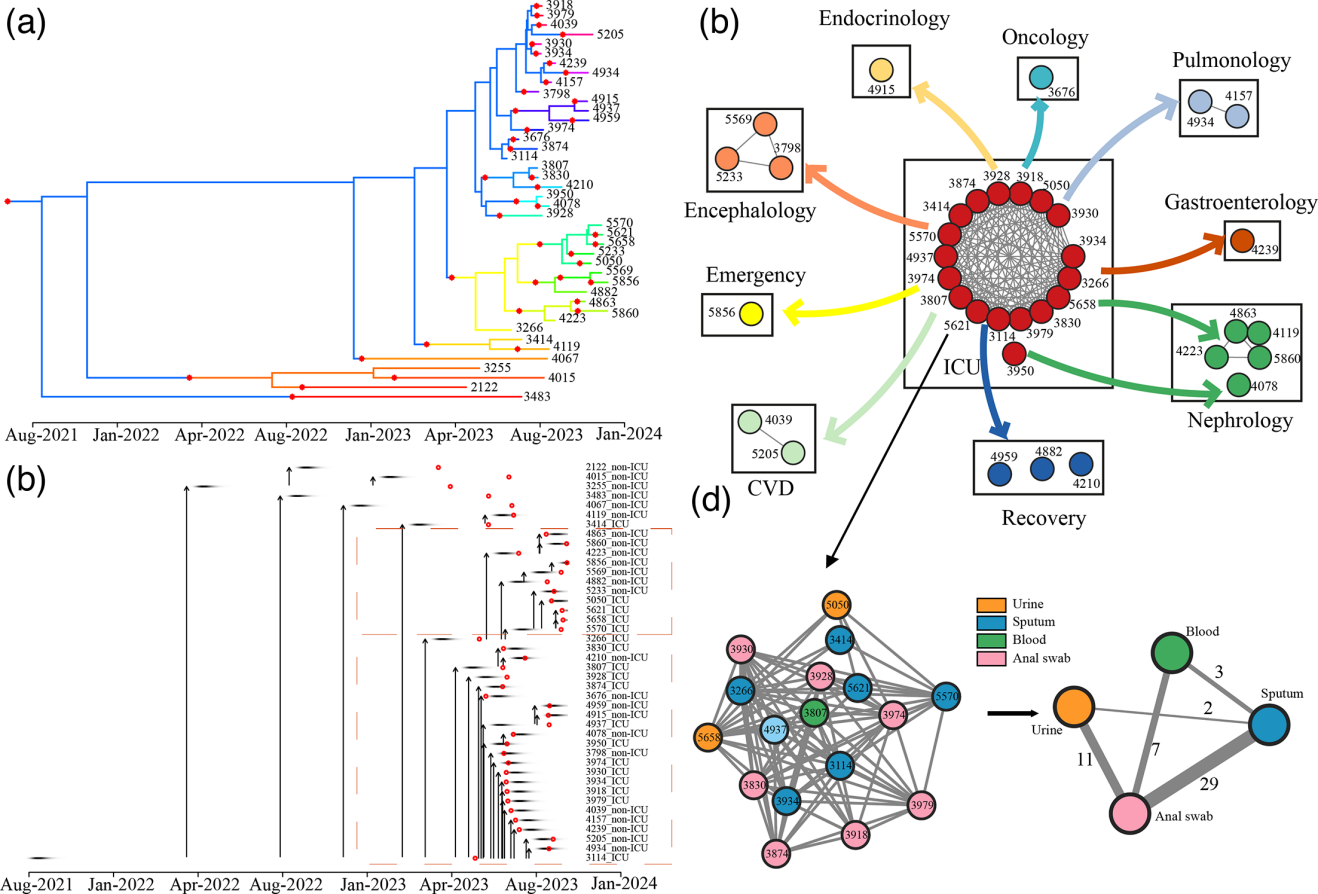
Inference transmission of ST11 CRKP during the hospital outbreak. (**a**) A coloured phylogeny of the 43 CRKP-ST11 isolates in this study. In the phylogeny, each branch was coloured separately for each patient. The colour changes indicate transmission from one patient to another. (**b**) Transmission of 43 CRKP-ST11 isolates. Each horizontal line represents an isolate, and the vertical arrows indicate the transmission from one patient to another. The collection date is represented by the red circle. (**c**) The transmission network is based on SNP distance (threshold=16). Each node represents an isolate, and the nodes are coloured by departments. The line indicates the transmission event. The significant protein mutation is labelled within the transmission. (**d**) A smaller transmission network indicates the CRKP circulation in the ICU. The nodes are coloured by sample sources. The weight of the edge indicates the number of transmissions, labelled near the edge as well. ICU: Intensive Care Unit; CVD: Cardiovascular disease; DEL: Deletion.

To further analyse the origin of the CRKP isolates, the coloured phylogeny and consensus transmission tree (Fig. 3a) showed that the outbreak started from ICU patient 3114 with multimorbidity in February 2021. It initially affected patients 3255 and 3483 from the oncology department, patient 4067 from the encephalology department, and patient 3414 from the ICU. Afterward, the inference indicated two main clusters of onwards transmission. These included a larger 3114-initiated cluster involving 23 patients and a smaller 5570-initiated cluster involving 11 patients, followed by several non-ICU routes of transmission, from patient 4882 to two patients (5569 and 4856) and from patient 4223 to two patients (5860 and 4863). The phylogenetic transmission indicated the ICU could be the origin of CRKP transmission in this study (Fig. 3b).

To further validate the ICU as the reservoir of CRKP transmission in the hospital, a SNP-based method was applied to investigate the transmission. After combining the SNPs with the data from the enrolled departments, we constructed the potential transmission routes on the departmental level (Fig. 3c). It is estimated that the CRKP ancestor might originate from ICU departments and then spread to the other nine departments. One separate transmission occurred from patient 3950 with diabetes and pulmonary disease to patient 4078 with cardiovascular disease in nephrology department. The low SNP distance in ICU isolates indicated a small clonal expansion in the ICU. Combined with the phylogenetic transmission, the ICU was proposed to be the reservoir and ancestor responsible for hospital outbreaks. To investigate the extent of isolate transmission within the ICU, we combined the SNP distance with the source group (Fig. 3d). We found that the isolates from the patient’s anal swabs were highly associated with sputum, with 29 transmission events identified (SNPs ≤16). Additionally, the CRKP isolates from UTI patients genetically close to those from patient anal swabs.

## Discussion

In this study, we reported an outbreak of CRKP and analysed the inter-host transmission within a hospital setting. The sampling of patients revealed ongoing colonization with *K. pneumoniae*. The ST11 CRKP isolates in our study exhibited a high number of resistance determinants in their genotypes and the findings highlight the emergence of increasingly serious threats in terms of antimicrobial resistance. ST11, an epidemic clone in China, has shown a propensity because of its ability to acquire different virulence-associated genes and transform into multidrug-resistant hvKP [36]. This study aligns with the findings of other studies on hospital-acquired infection, which have also reported persistent colonization by CRKP over an extended period [37,39]. Most of the ST11 CRKP isolates (95.3%, 41/43) in our study were found to harbour the *bla*_KPC-2_ and *bla*_TEM-1B_ genes. Additionally, most of the strains carried *bla*_CTX-M-65_ indicating a trend toward the convergence of antimicrobial resistance and virulence genes. A previous study documented the dissemination of the CRKP-ST11 clone co-producing *bla*_CTX-M-65_ and *bla*_KPC-2_ in the ICU and highlighted the hypermucoviscosity displayed by CRKP strains [40]. The recent emergence of ST11 CRKP strains causing severe infections and resulting in high mortality rates has been widely reported in multiple hospitals in China [10,41, 42]. More alarmingly, a fatal outbreak caused by ST11 CR-hvKP occurred in the ICU, resulting in a poor prognosis for all infected patients [43]. The notable increase in the prevalence of ST11 CRKP has led to concerns about virulence gene acquisition across diverse healthcare settings.

The CRKP strains of serotype KL64:O1/O2v2 in our study exhibited prolonged circulation within the ICU, with subsequent transmission to other healthcare-associated departments. Due to the scarcity of single rooms in the ICU, there was an elevated risk of nosocomial transmission of CRKP, which could be attributed to the practice of cohort nursing for colonized or infected patients in the ICU. The two major subclones of CRKP associated with hospital infection were CRKP-ST11 KL64:O1/O2v1 and KL47:OL101 [44]. In our study, the consistent nosocomial CRKP infection contributed to CRKP-ST11 KL64:O1/O2v1. Interestingly, no CRKP-ST11 KL47:OL101 strain was found at our hospital. Subclonal shifting in CRKP-ST11 strains has been reported in China starting since 2013 at a tertiary hospital [45]. Subsequently, a longitudinal and latitudinal study in China demonstrated the predominant KL64:O1/O2v1 replaced KL47:OL101 with the *recC* mutation, which enhances its pathogenicity [46]. Our hospital has experienced successful clonal shifting as evidenced by the emergence of CRKP-ST11 KL64 and the absence of KL47. Apart from CRKP-ST11, one hvKP from a cancer patient was an ST25 KL2:O1/O2v2. This strain is unique because it is the only one coharbouring *iroB* and *peg-344*, with phenotypic HM-positive and moderate biofilm formation ability. The first ST25-KL2 strain carrying *bla*_NDM-1_ was identified in Guangdong in 2013 [1] and a recent report of *bla*_NDM-1_ positive ST25 KP isolated from a neonate with sepsis showed high virulence with multiple organ damage in infected mice [47]. The limited reports and hypervirulence of CRKP-ST25 indicate the new tendency of emerging clonal expansion in China.

To investigate the high SNP count in non-ICU strains, a bacterial genome-wide association study (bGWAS) was conducted, identifying 17 SNP-associated genes that may drive the transmission of strains from ICU to non-ICU patients. Among these genes, those encoding transport functions were the most numerous, and they are involved in transporting amino acids, nucleotides, lipids, and carbohydrates. Previous studies on CRKP isolates have shown that mutations in transporter genes are a primary mechanism of fosfomycin resistance [48]. Beyond mutations related to transport in fosfomycin resistance, transporter genes such as peg344 are recognized as key virulence factors in hvKP [49]. The high mutation rate in transporter genes between CRKP strains from ICU and non-ICU patients suggests a change in virulence. A recent epidemiological study has indicated that ICU patients are at high risk for infection due to factors such as invasive procedures, immunosuppression, and advanced age [50]. A plausible explanation for the potential change in virulence is that patients admitted to the ICU often have compromised immune systems, making them more susceptible to colonization by CR-hvKP strains that exhibit decreased virulence in such environments.

In the present study, with the strong link of epidemiological connections, we used a cut-off value of 16 for SNP differences to estimate the potential transmission in the teaching hospital in China, as patients lacking epidemiological links can be well separated by 16 SNPs [32]. Based on the timeline, the transmission dynamics of KP-ST11 were observed to shift from the intra-ICU to inter-facility starting in 2021. This difference in transmission can be explained by the infection control measures taken in January 2022 and March 2022. The infection control measures in our hospital involved hand hygiene enforcement, environmental cleaning once daily, and mask-wearing. After those measures, *K. pneumoniae* infections in the ICU were well controlled with no cases in April 2022. To validate the transmission shift originating from the ICU, we used a combination of SNP-distance and TransPhylo methods to infer transmission within hospital facilities. While the SNP distance method has demonstrated its efficacy in predicting transmission pairs, the absence of certain epidemiological information, such as collection time and geographic location, can influence the accuracy of transmission prediction. False positive transmission pairs may arise when there is a lack of robust epidemiological data. In our study, since all the CRKP strains were collected within the hospital, the geographic location of all strains strongly correlated with the paired isolates. To fix the temporal gaps and genomic variations between transmission events of CRKP strains, TransPhylo utilizes a reversible jump Markov chain Monte Carlo (MCMC) algorithm to analyse genomic data with time labels. The model takes into account the observed cases and incorporates a branching process with a constant reproduction number throughout the outbreak. With the hybrid methods for transmission analysis, we revealed the detailed transmission route in the hospital at multiple levels including patient-to-patient, facility-to-facility, and source-to-source transmission. The results of the hybrid analysis prove valuable for identifying the most likely scenario for transmission events, which may be helpful for future transmission investigations. The findings not only clarified the ancestor of the CRKP outbreak as patient 3114 but also demonstrated the circulation of CRKP in the ICU with subsequent transmission to other departments. We also revealed that within the ICU, CRKP isolates obtained from anal swabs play a significant role in clonal spread. With this information, decolonization could be applied in hospital settings such as on the surface of public toilets, to reduce the prevalence of CRKP infection, as this outbreak strain exhibited resistance to multiple antibiotics, such as carbapenem, penicillin, and cephalosporin, and contains virulence genes encoding enterobactin, mucoid regulators, yersiniabactin, iron transporters, and adhesions. To prevent outbreaks, it is advisable to implement admission screening as a means of early detection of CRKP infection and for the enforcement of hand hygiene and environmental disinfection, as previously outlined [51].

The primary limitation of our study was the limited sample size collected within the hospital from 2020 to 2023. This was mainly due to the small size of the hospital and the COVID-19 restrictions that impacted hospital admissions. Another limitation is that all the isolates were collected only from patients without environmental sample collection in a single healthcare setting. To gain a deeper understanding of the transmission dynamics within hospitals, samples from the environment and healthcare workers should be included and isolates from multiple centres are necessary for a comprehensive genetic analysis. Additionally, long-read sequencing can be utilized to provide valuable insights into the complete characterization of carbapenemase-encoding plasmids. Furthermore, an appropriate animal model can be applied for the evaluation and identification and CRKP virulence ability.

In conclusion, our study revealed that CRKP transmission occurred at a teaching hospital in China and was characterized by genetic features. The CRKP strains involved in the hospital were all MDR bacteria that mainly harboured the *bla*_KPC-2*,*_*bla*_TEM-1B*,*_ and *bla*_CTX-M-65_ genes. For inference of transmission, we utilized the combined strategy of using the SNP distance-based method and a phylogenetic transmission tree to reveal the ICU is the reservoir of the outbreaks in the hospital. Furthermore, active screening programmes and infection control measures should be implemented to minimize the spread of CRKP infection within the hospitals, with particular emphasis on ICUs.

## supplementary material

10.1099/mgen.0.001299 Uncited Table S1.

## Abbreviations

BALF: Bronchoalveolar lavage fluid
CRKP: Carbapenem-resistant Klebsiella pneumoniae
CVD: Cardiovascular disease
ICU: Intensive care unit
MIC: Minimum inhibitory concentration
SNP: Single nucleotide polymorphism
ST: Sequence type
